# John Edward Fogarty: Where are you now that we need you—and from where will your successor come?

**DOI:** 10.3389/fpubh.2026.1944768

**Published:** 2026-10-08

**Authors:** Kerim M. Munir

**Affiliations:** 1 Department of Psychiatry and Department of Pediatrics, Harvard Medical School, Boston, MA, United States; 2 Division of Developmental Medicine and Department of Psychiatry and Behavioral Sciences, Boston Children's Hospital, Boston, MA, United States

**Keywords:** child and adolescent psychiatry, Fogarty International Center, global health, mental health policy, NIH, research capacity, Türkiye

## Abstract

John Edward Fogarty (1913–1967), known across Washington as “Mr. Public Health,” was the bricklayer-congressman who, with Senator Lister Hill, helped build the modern National Institutes of Health (NIH); he died on the morning of his fourteenth swearing-in. The Fogarty International Center (FIC), established in his memory in 1968, became a principal instrument for developing global health research capacity, training some 6,000 scientists from more than 100 nations. Born in the infectious-disease emergencies of its first era—HIV/AIDS above all—FIC deliberately widened its scope at the turn of the century to the noncommunicable diseases of low- and middle-income countries—cardiovascular disease, tobacco-related illness, and chronic conditions across the lifespan, together with mental health and neurodevelopmental disorders; within that paradigm shift, FIC’s collaboration with the National Institute of Mental Health made possible the program described here. Drawing on a principal investigator’s twenty-five-year account of FIC’s first award in Türkiye—where, in the aftermath of the 1999 earthquakes, FIC-supported research training developed alongside the country’s first National Mental Health Policy—this Perspective revisits Fogarty’s legacy as the Center again faces proposed elimination, and argues that where public trust has been strained, the remedy is accountability and reform, not the elimination of the nation’s critical global scientific arm.

## 1 Introduction

On the morning of January 10, 1967, hours before he was to be sworn in for his fourteenth term, Congressman John Edward Fogarty of Rhode Island died suddenly of a heart attack in his office. He was 53. The House of Representatives, which ordinarily meets and adjourns briefly upon the death of a member, convened as scheduled that day—swearing-in sessions are mandatory—and so the chamber took its oaths in the shadow of the man known across Washington as “Mr. Public Health.” The tributes came swiftly. Howard Rusk, the influential physician-journalist, wrote in the *New York Times* that no one in the nation’s history had done more to promote better health services, health facilities, and health research than Representative Fogarty (1, 2). President Johnson, who a year earlier had presented Fogarty with the American Heart Association’s Heart-of-the-Year Award, called him “a major architect in the fields of health and education” (3). Historians of the period have speculated that had he lived, the United States might have achieved universal health coverage (4).

More than four decades ago, Leon Eisenberg asked, “Rudolf Ludwig Karl Virchow, where are you now that we need you?”—a summons for medicine to reclaim its moral and social mission (5, 6). Virchow, who founded cellular pathology while insisting that medicine is a social science and that politics is nothing but medicine on a grand scale, was invoked by Eisenberg against a biomedicine grown indifferent to the social determinants of its own findings. The same question must now be asked of John Fogarty. In an era of fractured politics and resurgent isolationism in global health, his example reminds us of what it means to govern with vision, to lead with humility, and to invest in the health of all.

## 2 A builder of institutions

Fogarty became an apprentice bricklayer upon finishing high school, following his father and older brother into the trade. By 24 he had been chosen president of his local Bricklayers Union; at 27 he was elected to the United States House of Representatives as a New Deal Democrat from Rhode Island. As chairman of the House Appropriations Subcommittee for Labor, Health, Education, and Welfare—from 1949 to 1953, and again from 1955 until his death—he forged with Democratic Senator Lister Hill of Alabama the legislative partnership that built the modern National Institutes of Health (4, 7). When Fogarty entered Congress in 1941, the entire federal investment in medical research stood at roughly $3 million (1); the NIH appropriation itself grew from $37 million in 1949 to $1.24 billion by 1967—a more than thirtyfold expansion in under two decades (8, 9). In 1959, Fogarty and Hill received special Lasker Awards for extraordinary public service, a distinction rarely conferred on those outside the profession. Fogarty had no axe to grind: “Nothing happened to me as a kid that made me decide medicine must be important” (1); he simply understood, earlier and more completely than his contemporaries, that federal investment could transform a nation’s health.

In the bipartisan tradition inaugurated by Hill and Republican Senator Harold Burton in the Hospital Construction Act of 1946, Fogarty and Hill secured funding to construct and expand medical, dental, pharmacy, and nursing schools, ensuring that science and service advanced together (8). Fogarty championed the Community Mental Health Centers Act amendments and the expansion of teaching and research for citizens with intellectual disability; he sponsored the Older Americans Act of 1965, creating the Administration on Aging; he advanced vocational rehabilitation, educational access for deaf and blind children, library services, environmental health, and the National Foundation on the Arts and the Humanities (8). In his freshman year, some 12,000 disabled persons were rehabilitated into employment under the public program; by 1967 the figure was 154,000 (1). And he was unafraid of presidents of his own party: in 1961 he publicly accused President Kennedy of dealing a “devastating blow” to cancer research by trimming $60 million from the NIH budget, then led the congressional effort to restore it; in 1965, as President Johnson signed the Health Research Facilities Act at the NIH, Fogarty told him, “We would have had more if you hadn’t cut the funds” (4).

## 3 Working across the aisle

Fogarty’s greatest legislative successes came through the aisle he crossed. His closest House ally was Melvin Laird, the Wisconsin Republican who later served as Secretary of Defense. Laird would remember inviting Fogarty to dinner on the evening before that final swearing-in—the eve, as it proved, of his death; Fogarty had reluctantly declined: his desk was piled high, and he preferred to stay late clearing his files, preparing for the new session, and catching up on his work. They would grab a bite, he promised, and celebrate after the ceremonies the next day (10). Theirs was more than friendship; it was a conviction that public health is a nonpartisan priority. It was Laird who delivered Fogarty’s eulogy, remembering a colleague who asked not one’s party but only whether one was doing right by the health of the people (10, 11). That ethos carried landmark health legislation through the Eisenhower, Kennedy, and Johnson administrations—and it feels almost anachronistic today.

## 4 A champion of global health before its time

Long before “global health” existed as a discipline, Fogarty understood that the health of Americans could not be protected in isolation from the rest of the world. He attended nearly every session of the World Medical Association, co-sponsored with Hill the “Health for Peace” legislation of 1958 and dreamt of an institution where the best scientists from around the world would collaborate on the hardest problems of human health. He did not live to see it. On July 1, 1968, President Johnson issued the Executive Order establishing the John E. Fogarty International Center for Advanced Study in the Health Sciences at the NIH—not merely a tribute, but a continuation of his vision (8).

In the nearly six decades since, the Fogarty International Center (FIC) has served as the bridge between the NIH and the global health community. Its story has unfolded in distinct eras, each led by a director who met the emergency of his time and left the Center larger than he found it. Its early contributions included the international conferences that stimulated the worldwide campaign to eliminate polio and expand childhood immunization. The defining emergency of the first era arrived with HIV/AIDS: with the creation of the AIDS International Training and Research Program (AITRP) in 1988, FIC became a principal mechanism for building sustainable research capacity in low- and middle-income country (LMIC) settings (12). Philip E. Schambra (director, 1989–1998) met that emergency head-on, persuading lawmakers of the Center’s critical value and channeling the resulting budget growth into an aggressive expansion of AITRP—building the laboratory and field infrastructure through which investigators in LMICs could study, and their health systems could deploy, modern HIV/AIDS therapies (12). On that AIDS-built foundation, Gerald T. Keusch (director, 1998–2003) undertook a deliberate widening of FIC’s intellectual scope: recognizing the epidemiological transition then gathering across LMICs, he launched research training programs in brain disorders, tobacco, stigma, international research ethics education and curriculum development, and cardiovascular and other chronic diseases, and brought the Disease Control Priorities Project to FIC—the blueprint for today’s holistic global health research (12, 13). Within that NIH-wide broadening, the National Institute of Mental Health’s collaboration with FIC—the International Collaborative Operational Health Research Training Award (ICOHRTA) and Non-Communicable Diseases across the Lifespan (NCD-LIFESPAN) lineage—carried mental health and neurodevelopmental conditions, newly counted among the grand challenges of global health, into the Center’s portfolio (14). The era that followed consolidated the enterprise: Roger Glass, the longest-serving director, who led FIC from 2006 to 2023, and Peter Kilmarx, his deputy and successor as acting director—both highly experienced infectious disease physicians—came to frame research capacity itself as the world’s readiness for the next pandemic (15).

Across these eras, FIC’s programs have provided research training for some 6,000 health scientists from more than 100 nations, including about 1,000 Americans; many have become national scientific and global health leaders (12). As LMIC research programs matured, the benefits became bidirectional: FIC trainees helped contain outbreaks, respond to global emergencies, strengthen health security, research ethics and responsible conduct of research, and open local scientific opportunities that could not be pursued elsewhere (12). In this the Center was not departing from its namesake’s vision but completing it: Fogarty’s “Health for Peace” was a health-for-all ideal, and the Center—small, generative, drawing the institutes of the NIH, from mental health to heart, lung, blood, and cancer, into partnerships abroad—became a mechanism that integrates the missions of the entire NIH into one global health vision, and a hub within a wider global research network (8, 12).

## 5 The Fogarty ethos in the field: a personal witness

I can attest to what John Fogarty’s posthumous institution accomplishes, beyond infectious diseases, for I have spent twenty-five years within it working on mental health and developmental disabilities research and training. The program which I directed at Boston Children’s Hospital received its first NIH funding in the fall of 2000—months after two catastrophic earthquakes struck northwestern Türkiye (Turkey, as it was then named)—official counts recorded more than 18,000 deaths in the Marmara event, and 850 in the subsequent Düzce event, with contemporaneous accounts estimating total mortality above 25,000 once the unrecovered were counted laying bare how heavily the country’s mental health services were concentrated in its major cities, with little integrated into primary care in the affected provinces (16). In the emergency’s immediate aftermath, I served as an international program officer for health and psychosocial programs with UNICEF in Türkiye—a liaison role with the Ministry of Health under the Recovery Plan for Turkish Children that brought me into the country’s post-disaster mental health planning and from which the work that follows grew (16). The FIC ICOHRTA (non-HIV/AIDS) award and its successor program in noncommunicable diseases across the lifespan (NCD-LIFESPAN) descended directly from the Keusch-era lineage described above and, innovatively, brought mental health research training and research ethics within its competitive scope. With Turkish colleagues, this seed program helped encourage a merit-based approach to research and training. The Republic of Türkiye’s first National Mental Health Policy—developed under World Bank sponsorship after the earthquakes, organized around integration with primary care and containing, distinctively, an integral child and adolescent mental health policy module developed with the national child and adolescent psychiatry association—was published by the Ministry of Health in 2006 (16, 17). In 1995, there were 35 child and adolescent psychiatrists in the entire country, trainees included—as the International Association for Child and Adolescent Psychiatry and Allied Professions (IACAPAP) Bulletin’s title put it, “only 35 for ten million children and adolescents” (18). By 2011 the Ministry of Health counted 206 specialists (19); by 2013 there were more than 400; and by 2025, on the estimate published in the same source’s retrospective note, some 1,200 physicians had completed or entered specialty training—an expansion, however counted, of well over an order of magnitude within a single generation (18). By 2012, Türkiye was also independently ranked first among all LMICs in the scientific output of child mental health research (20). The chronology documented here was, in part, a strategic opening and, in part, serendipity. The growth had many parents: the Ministry of Health, which converted policy into staffing norms and residency positions; the nationwide expansion of universities and their medical faculties; and the devoted labors of the country’s national country’s national adult psychiatry, child and adolescent psychiatry, psychology, and counseling and guidance associations. What can be documented is a sequence: a disaster seized as a window of opportunity (16); an FIC award that modeled, albeit in a limited way, research training at a moment when mentoring capacity was scarce; and a national policy, developed in part by the same colleagues, that gave mental health a free-standing place in national health planning (16, 17).

What the FIC award provided, albeit limited, was upstream and specific: it set an example of merit-based selection of research trainees in affiliate institutions; structured training in research methods and research ethics; mentored progression to peer-reviewed publication; and a design that leveraged a small number of senior academic mentors across successive cohorts, so that scarce mentorship seeded a mentoring culture rather than becoming its ceiling. What FIC did not and could not provide—the World Bank financing of post-earthquake reconstruction, the Ministry of Health’s policy implementation, the nationwide expansion of universities and residency positions, and the sustained advocacy of the national professional associations—were the larger engines of workforce growth.

Multiply that story across a hundred nations and six thousand trainees in many fields, and the scale of the return on John Fogarty’s vision of global health as a collaborative, interconnected enterprise becomes clear. When the Czech Republic joined the European Union in 2004—thereby graduating from eligibility for FIC support—I asked Aron Primack, long an influential program officer in FIC’s Division of International Training and Research, whether Fogarty could take credit. He smiled: “Yes, of course.” The exchange is offered not as a causal claim—the Czech accession had many authors—but as a personal recollection of how some within FIC understood the wider diplomatic meaning of scientific cooperation: a conviction, traceable to John Fogarty’s own “Health for Peace,” that American support for medical research abroad returns dividends in goodwill as well as in health.

## 6 A legacy under threat—and a call to action

Funding figures below refer to the NIH discretionary program level unless otherwise noted; the legislative status is described as of September 2, 2026. Last year, FIC faced the most serious threat in its history. The administration’s fiscal year 2026 initial budget request proposed cutting the NIH by roughly 40 percent, consolidating its 27 institutes and centers into eight, and eliminating FIC outright—proposals accompanied by sweeping reductions in the NIH workforce and a 15 percent cap on the indirect-cost reimbursement rate for research grants that drew legal challenges from nearly two dozen states (21). Congress refused. In the FY2026 omnibus signed into law on February 3, 2026, a bipartisan Congress funded the NIH at $47.2 billion—a $400 million increase over the prior year—preserved the FIC at $95 million, wrote hundreds of program-level funding amounts into statute, and required notification before changes to NIH grants (21–23).

The reprieve lasted two months. On April 3, 2026, the FY2027 budget request returned to the same well: $41 billion for the NIH—a reduction of roughly $5 billion, or 12 percent—with the FIC again slated for elimination, alongside the National Institute on Minority Health and Health Disparities and the National Center for Complementary and Integrative Health, and a renewed 15 percent indirect-cost cap that Congress has repeatedly rejected (24, 25). The damage is not merely prospective; some of it has already been done. Even the enacted FY2026 appropriation took nearly two months to reach the NIH, delaying award issuances, and the administration has terminated or frozen thousands of grants where it could, notwithstanding congressional direction (25–27). When elimination of FIC was first proposed in 2017, the global health community’s response was unequivocal: preserving the Center benefits Americans and the world alike, and its closure would threaten not only global health but the medical research enterprise in the United States (28, 29). Lawrence Gostin, an authority in public health law, human rights, and global health policy, called the Center, simply, “a national treasure” (30). Its entire budget—$95 million—is two-tenths of 1 % of the NIH; eliminating it would save almost nothing and forfeit a training pipeline many LMICs—and the United States global research enterprise -- had come to rely on.

In recent weeks, the pattern has begun to repeat itself. On June 9–10, 2026, the House Appropriations Committee approved its FY2027 Labor-HHS bill, providing a $47.3 billion NIH base budget—a modest increase—and funding all 27 NIH institutes and centers, thereby rejecting, for a second consecutive year, the proposed elimination of FIC (31). As of this writing, a continuing resolution signed into law on September 2, 2026 (Public Law 119–103) funds the government—the FIC included—at FY2026 levels through December 11, 2026, deferring the FY2027 decision to the year-end; the Senate Appropriations Committee has yet to agree on topline levels or report any of its twelve bills (31, 32). The courts, meanwhile, have spoken: a federal district court permanently enjoined the administration’s 15 percent indirect-cost cap in April 2025, a ruling the First Circuit affirmed on January 5, 2026, and Congress has kept the statutory prohibition on modifying the policy in force (33). The administration justifies its proposals by asserting that the NIH “broke the trust of the American people with wasteful spending, misleading information, risky research, and the promotion of dangerous ideologies” (25). If trust has indeed been strained, the remedy is accountability and reform—not the elimination of the nation’s global scientific arm. Congress, by statute, and the courts, by judgment, have, in effect, said the same.

To return to Eisenberg’s summons: these are not merely budgetary choices; they are moral ones. They reflect a narrowing conception of national interest and a refusal to recognize that pandemics, antimicrobial resistance, noncommunicable diseases and mental health, as well as climate-driven health crises, do not respect borders. The Institute of Medicine—now the National Academy of Medicine—held a generation ago that the direct interests of the American people are best served when the United States acts decisively to promote health around the world, leading from its strengths in medical science and technology, and that failure to engage would diminish America’s stature and jeopardize our own health, economy, and national security (33). The economics are equally plain: the total U.S. investment in smallpox eradication alone is returned to the United States every 26 days—an annual dividend of more than fourteenfold (33). John Fogarty understood all this six decades ago. President Johnson affirmed it in 1966, honoring Congressman Fogarty at the White House: “No society can be great which is not first of all healthy,” adding that “the healing miracles that we achieve must not be gifts for Americans only” (3).

The congressional response has been, quite literally, the Fogarty–Laird model in action. The FY2026 rescue was engineered by the Senate Appropriations Committee under its chair, Susan Collins of Maine—the first Republican woman to lead the committee, whose stated priorities on taking the helm included “funding lifesaving biomedical research”—working with her Democratic vice chair, Patty Murray of Washington, through a bipartisan partnership both have credited publicly, and which also steered the government out of a 40-day shutdown in November 2025 (22, 34). A Republican chair and a Democratic vice chair, jointly protecting the NIH: John Fogarty and Melvin Laird would have recognized the arrangement immediately. Congress must now hold that line for FY2027—reject the proposed cuts, safeguard the NIH and the FIC, and reassert the United States’ leadership in global research and research education. Protecting public health has never rightly been a partisan question.

## 7 Discussion: the road not taken

John Fogarty never took his fourteenth oath. The House swore in its members that January morning without him. The measure of what was lost is best taken in what he built: a bricklayer’s son who laid, brick by legislative brick, the America’s foundations of modern laboratories, medical schools, hospitals, and institutions for the mentally ill and the intellectually disabled—and who understood, before almost anyone, that those foundations could not stop at the water’s edge.

The capacity-building model FIC pioneered is not beyond criticism. Systematic reviews of health research capacity development in LMICs find uneven evidence on sustainability and note persistent risks: dependence on external funding cycles, emigration of trained investigators, inequities in authorship and agenda-setting, and the asymmetries inherent in North–South partnership (35, 36). These critiques argue for reforming how capacity is built—longer horizons, institutional rather than solely individual investment, and genuine local ownership of a collaborative global research agenda—not for abandoning the enterprise; indeed, they presuppose the continued existence of the very institution they seek to improve. FIC’s own history—widening, era by era, from infectious disease to the whole breadth of global health—is the record of a Center capable of precisely such learning. That is precisely the distinction between accountability and elimination on which this Perspective’s argument rests.

If John Fogarty were with us today, he would urge not retreat but renewal. He would remind lawmakers that bipartisan collaboration is a sign of strength, not weakness, and remind the scientific community that its greatest measure lies not in publications or patents but in the lives it helps save and makes healthier. John Fogarty’s own words guide not only the Center that bears his name as the memorial, but the NIH itself, which bears his imprint as the monument: “The nations of the world can and must share their knowledge and other resources so that people everywhere may have the blessing of better health, and through health, may move forward to new levels of peaceful productivity” (8). John Edward Fogarty, where are you now that we need you—and from where will your successor come?

## Data Availability

The original contributions presented in the study are included in the article, further inquiries can be directed to the corresponding author/s.
